# Significant reduction in abundance of peridomestic mosquitoes (Culicidae) and *Culicoides* midges (Ceratopogonidae) after chemical intervention in western São Paulo, Brazil

**DOI:** 10.1186/s13071-020-04427-1

**Published:** 2020-11-07

**Authors:** Mikel A. González, Erin Dilger, María M. Ronderos, Gustavo R. Spinelli, Orin Courtenay, James G. C. Hamilton

**Affiliations:** 1 Division of Biomedical and Life Sciences, Faculty of Health and Medicine, Lancaster University, Bailrigg, LA1 4YG Lancashire UK; 2 Present Address: Departamento de Sanidad Animal, Instituto Vasco de Investigación y Desarrollo Agrario (NEIKER), Derio, Bizkaia Spain; 3 Zeeman Institute and School of Life Sciences, Gibbet Hill Campus, University of Warwick, Coventry, CV4 7AL UK; 4 División Entomología, Museo de La Plata, Paseo del Bosque s/n, 1900 La Plata, Buenos Aires Argentina

**Keywords:** Abundance, Brazil, Culicidae, *Culicoides*, Insecticide intervention, Distribution, Lambda-cyhalothrin, Species composition, Chickens

## Abstract

**Background:**

We assessed the impact of two sand fly insecticide interventions (insecticide spraying and insecticide-impregnated dog collars) on the peridomestic abundance and distribution of mosquitoes (Culicidae) and biting midges (Ceratopogonidae) in western São Paulo (Brazil) in a long-term (42-month) evaluation. Both of these dipteran groups are vectors of diseases of medical and veterinary relevance to humans and domestic animals in Brazil.

**Methods:**

The interventions in the 3-arm stratified randomised control trial were: pheromone + insecticide (PI) (chicken roosts were sprayed with microencapsulated lambda-cyhalothrin; pheromone lure has no effect on the Diptera pests studied here); dog-collars (DC) (dogs fitted with deltamethrin-impregnated collars); and control (C) (unexposed to pyrethroids) were extended by 12 months. During that time, adult mosquitoes and midges were sampled along 280 households at three household locations (inside human dwellings, dog sleeping sites and chicken roosts).

**Results:**

We collected 3145 culicids (9 genera, 87.6% *Culex* spp.) distributed relatively uniformly across all 3 arms: 41.9% at chicken roosts; 37.7% inside houses; and 20.3% at dog sleeping sites. We collected 11,464 *Culicoides* (15 species) found mostly at chicken roosting sites (84.7%) compared with dog sleeping sites (12.9%) or houses (2.4%). Mosquitoes and *Culicoides* were most abundant during the hot and rainy season. Increased daytime temperature was marginally associated with increased mosquito abundance (*Z* = 1.97, *P* = 0.049) and *Culicoides* abundance (*Z* = 1.71, *P* = 0.087). There was no significant association with daily average rainfall for either group. Household-level mosquito and midge numbers were both significantly reduced by the PI intervention 56% [incidence rate ratio, IRR = 0.54 (95% CI: 0.30–0.97), *P* ≤ 0.05] and 53% [IRR = 0.47 (95% CI: 0.26–0.85), *P* ≤ 0.05], respectively, compared to the control intervention. The abundance of both dipteran groups at dog sleeping sites was largely unaffected by the PI and DC interventions. The PI intervention significantly reduced abundance of mosquitoes inside houses (41%) and at chicken roosting sites (48%) and reduced midge abundance by 51% in chicken roosting sites.

**Conclusions:**

Sprayed insecticide at chicken roosting sites reduced the abundance of mosquitoes and midges at the peridomestic level while dog collars had no effect on numbers for any group.
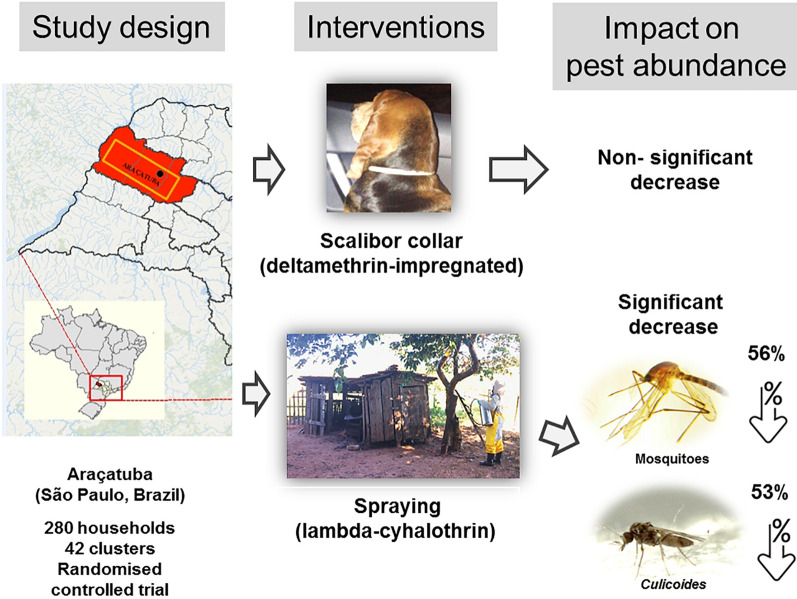

## Background

In Brazil, mosquitoes (Diptera: Culicidae) are by far the most important and well-studied group of blood-sucking insects [1] and > 450 species have been described [2]. Some pathogens transmitted to humans, wildlife, and domestic animals have the potential to cause significant morbidity and mortality [3]. *Aedes aegypti* is the vector of several viruses, most notably dengue, yellow fever, chikungunya, Zika and the filarial roundworm *Wuchereria bancrofti* which causes lymphatic filariasis [4]. *Culex quinquefasciatus* transmits the heartworm *Dirofilaria immitis*, causing microfilariasis in some coastal cities [5] and is incriminated in the transmission of several viruses such as Saint Louis encephalitis virus, Mayaro virus, eastern equine encephalitis virus and Rocio viral encephalitis virus [3]. *Culex* spp. also cause significant discomfort and allergic responses through their nocturnal nuisance biting activity and present an increased risk of transmission of new arbovirus and pathogens from avian hosts to humans [6].

The genus *Culicoides* (Diptera: Ceratopogonidae) includes almost 150 species of biting midges in Brazil [7] and are responsible for the transmission of several viral diseases such as Oropouche virus (OROV), which affects humans in the Amazon Basin, and bluetongue virus (BTV), which affects wild and domestic ruminants worldwide [8, 9]. OROV is one of the most common human arbovirus infections in Brazil and more than 30 major outbreaks and half a million cases have been reported since it was first isolated in 1955 in Trinidad and Tobago [10]. *Culicoides* species can transmit avian haemosporidians, particularly some species of the genus *Haemoproteus* [11]; however, the role of the biting midges as vectors of these parasites remain largely unknown in South-Central America. *Culicoides* midges, e.g. *C. paraensis*, cause a significant biting nuisance because of population size and their persistent biting activity [8, 12].

Sand flies (Diptera: Psychodidae) are also widespread in Brazil and are found in the same peridomestic environment as mosquitoes and biting midges. There are approximately 285 sand fly species in Brazil and 13 of these are proven vectors of *Leishmania* spp. [13]. *Lutzomyia longipalpis* is the most widespread and important vector of the protist parasite *Leishmania infantum* (Kinetoplastida: Trypanosomatidae), which causes visceral leishmaniasis (VL) in humans and dogs [14].

The recommended VL control options in Brazil include the reactive application of insecticides in houses and animal sheds to reduce vector numbers, the euthanasia of seropositive domestic dogs, the diagnosis and treatment of human cases, and public education [15, 16]. However, despite the efforts of the Ministry of Health, the burden (calculated from the mortality, morbidity, and disability) of VL in Brazil more than doubled between 1990 and 2016 [17].

Recently, a new vector control approach using both a lure of the synthetic version of a *Lu. longipalpis* sex pheromone (9-methylgermacrene-B) and spraying of microencapsulated lambda-cyhalothrin to reduce vector densities and canine *Leishmania infantum* infection incidence in dogs, was tested in a large-scale, long-term stratified randomised control trial (sRCT) in the Araçatuba region of western São Paulo State, Brazil. The trial which also investigated the use of Scalibor^®^ deltamethrin-impregnated dog collars, an established sand fly control device, was carried out in 33 municipalities and 9 districts of Araçatuba [18].

As part of this study, we investigated for the first time the impact of the two insecticide-based interventions (sprayed residual insecticide and insecticide-impregnated dog collars) on two biting dipteran groups, mosquitoes and *Culicoides* biting midges, which are pests often found in abundance in chicken sheds, other animal shelters, and inside human dwellings throughout Brazil [1, 3, 7–9, 12] along with *Lu. longipalpis* sand flies. In addition, the study also gave us the opportunity to assess the species richness, abundance, distribution, annual dynamics and influence of climatic conditions (temperature and rainfall) on mosquitoes and *Culicoides* midges in households.

## Methods

### Study area

**Fig. 1:**
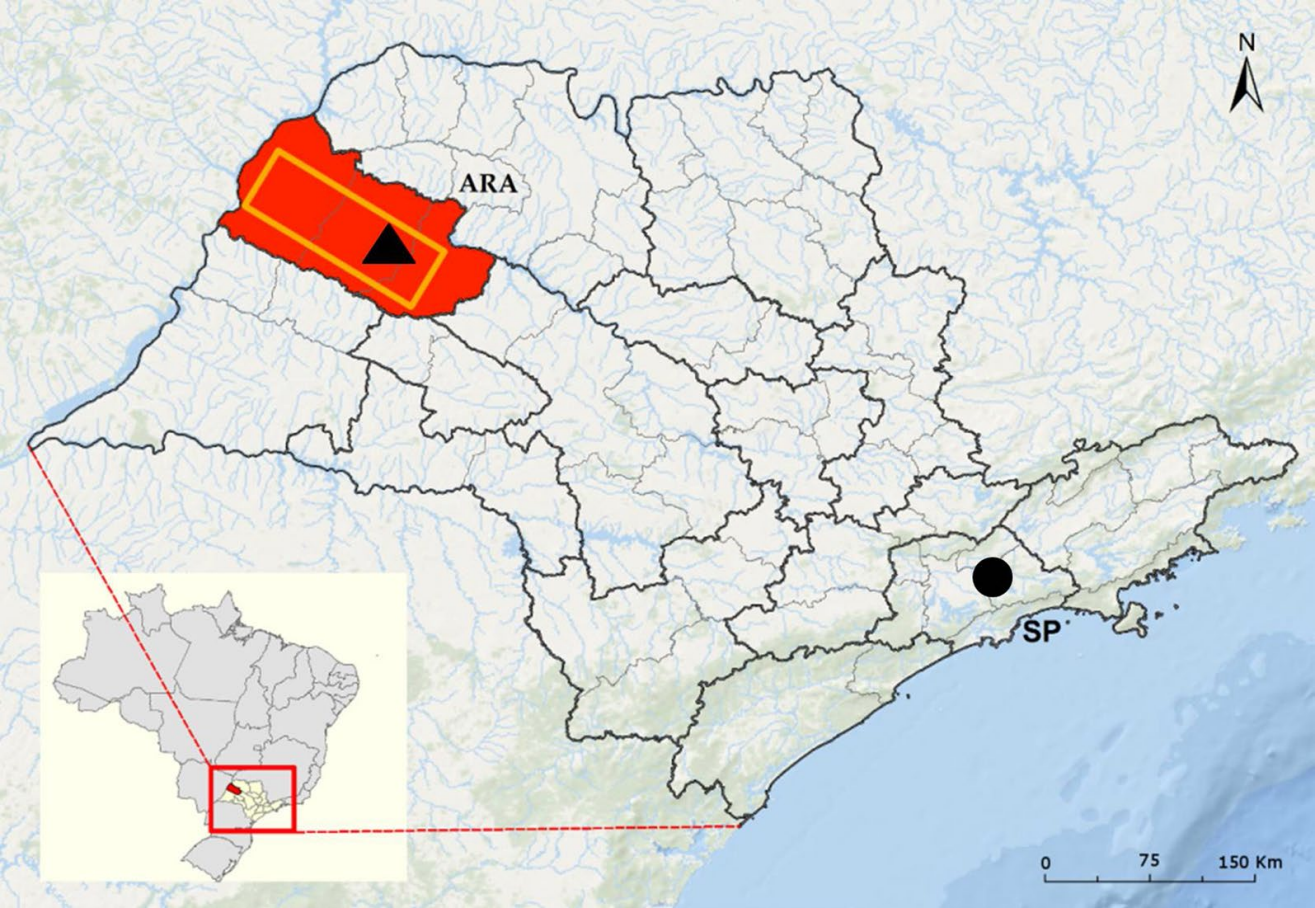
Map of the study area in São Paulo state, Brazil. The region of study (11,250 km^2^) is shown in an orange rectangle located within the mesoregion of Araçatuba (red coloured area). The location of Araçatuba city is denoted by a black triangle and the location of São Paulo city is denoted by a black circle (ArcGIS 10.4.1; layer sources: IBGE - Instituto Brasileiro de Geografía e Estatistica/Ocean Basemap)

Studies were conducted in the mesoregion of Araçatuba (21°20′89ʺS, 50°43′28ʺW; ca. 11,250 km^2^ and ca.700,000 inhabitants) in northwest São Paulo State, Brazil. A total of 280 households in 42 sRCT clusters were included in the Araçatuba region (Fig. 1, Additional file 1: Table S1). The climate in this region is the Aw type (tropical sub-warm and sub-dry) according to the Köppen-Geiger classification [19] with two distinct seasons; a dry and cool season from April to September (autumn through to winter), and a hot and wet season from October to March (spring through to summer). The mean annual temperature was 23.8 °C (min: 17.0 °C, max: 30.6 °C), total annual rainfall was 1309 mm, and the wettest months were January, February and December in decreasing order of rainfall (2014–2016). Climate data (rainfall and temperatures) were also obtained from a weather station located at Araçatuba city from July 2015 to April 2016 [20]. This station was selected to be representative for the 42-clusters studied (the farthest cluster was located 110 km away from the station in straight line).

All experiments were carried out within private households and within their yards either at the front or back of the house. The average number of hosts per household was (range; mean ± SD): dogs (1–12; 2.65 ± 1.80), chickens (1–125; 24.51 ± 21.80) and humans (0–10; 3.50 ± 1.83). Other poultry (geese, guinea fowl and ducks) and other animals (pigs and goats) were common and kept within the yard which may also have contained fruit trees, flowers or shrubs.

### Study design and trapping

The study design followed that of the previously described sRCT [18, 21] and collections of mosquitoes and biting midges were concurrently made when collecting sand flies. Clusters, households, and dogs were recruited in a three-step procedure (recruitment, cluster stratification, and randomisation and treatment allocation) [21].

The collections were made in each of the three arms of the trial: (i) synthetic pheromone + insecticide co-located in chicken roosting sites including chicken sheds (PI-arm); (ii) deltamethrin-impregnated collars fitted to dogs (DC-arm); and (iii) a placebo control (C-arm).

Within the PI-arm, microencapsulated lambda-cyhalothrin was sprayed using a hand-compression sprayer (GUARANY 441-10 compression sprayer; Guarany Industria e Comercio Ltda, São Paulo, Brazil) according to the guidelines of the Brazilian Ministry of Health of São Paulo State [15]. The pheromone lure containing 10 mg of synthetic pheromone for sand fly attraction, is known to be highly specific, with no attraction even to other subspecies of *Lu. longipalpis* sand flies [22]; therefore, we excluded any effect on mosquitoes and biting midges. Sprayed sites were mostly (i) variable size (open, closed, semi-closed) chicken sheds, (ii) roosting trees from ground level to 3 m up the roosting tree particularly on roosting branches, and into a lesser extent (iii) on walls adjacent to ground roosting chicken or similar unusual sites (3 m^2^ area).

Within the DC-arm, each dog living in the dwelling was provided with a collar impregnated with 1.0 g of deltamethrin (Scalibor^®^ Dog Collar, Intervet Productions S.A., France). Collars were replaced every 5–6 months across the study as needed according to the manufacturer instructions.

Control-arm (C), chicken shelters were sprayed with pure water (in the same manner as PI-arm) rather than insecticide, and dogs received a placebo collar. Households selected for the C-arm were described as insecticide-free by the householders as they had no previous residual insecticide application.

The study (42-month) was divided in rounds concurring the time to complete the insecticide application in the PI arm and the water spraying in the C arm. The applications were carried out in three monthly periods between January 2012 to March 2016 giving a total of 17 applications (four rounds per year).

Thus, in this present study we evaluated the impact of the residual insecticide lambda-cyhalothrin biting midges in the chicken roosts, dog sleeping places, and the interior of people’s houses (inside dwellings).

### Sampling

Adult mosquitoes and *Culicoides* biting midges were collected with CDC suction traps (HP Biomédica, Minas Gerais, Brazil) employing a standard incandescent bulb, and adapted to be powered by a rechargeable 6V battery [23]. Traps were attached to a fine mesh collecting bag with double ring. Trapping rounds were implemented for one day per round per household during a period of 18 h (set up in the afternoon and retrieved the following morning) approximately every three months after the lambda-cyhalothrin or deltamethrin-dog collar application. Each new round where trapping took place is referred to as a trapping round. After 13 rounds of insecticide intervention, we started four trapping rounds for both biting dipteran groups (round 14: 20 July - 10 August 2015; round 15: 15 October - 5 November 2015; round 16: 12 January - 27 February 2016; and round 17: 11 April - 3 May 2016), keeping insecticide interventions unaltered until the end of April 2016. Thus, the final dataset was generated from 123, 110 and 112 trapping days in 103, 88 and 89 households in 14, 12 and 13 intervention clusters in C-arm, PI-arm and DC-arm respectively, for each Diptera group.

The three CDC traps per household were one located close to a chicken roosting site (e.g. chicken shed or roosting tree), one at the dog sleeping site (e.g. a dog pen or kennel), and one within the house (e.g. a living room, kitchen or bathroom, to minimise disturbance of the residents). In the infrequent event of heavy rain or strong wind, the days’ collections were discarded, and trapping was repeated the following day.

### Sample processing and species identification

The live collected insects were placed in a − 20 °C freezer for 20 min to kill them prior to being placed in 70% ethanol. They were stored until the culicids were separated from *Culicoides* spp., sorted by sex and counted under a binocular stereomicroscope (Quimis Ltda., Sao Paulo, Brazil) at 4× magnification.

In Culicidae, female morphological features were not conclusive because of their preservation in alcohol. Male culicids were identified to species level based on male genitalia morphology. Because of the large numbers of *Culex* specimens, only a subsample (ca.30% of the total catches) were randomly selected from the three household locations and slide-mounted for determination of species. Heavy-sclerotized male genitalia was first cleared (10% potassium hydroxide for 24 h), then dehydrated (ethanol series from 70% to 100%) and finally immersed in a clearing agent (eugenol) before being mounted in Canada balsam and allowed to dry at room temperature for several days (adapted from Consoli & Lourenço-Oliveira [1]). Specimens were identified in the Laboratorio de Transmissores de Hematozoários of the Institute Oswaldo Cruz (IOC, Rio de Janeiro, Brazil) using taxonomic keys [3, 24–27]. *Culicoides* species identification was based initially on wing pattern and then confirmed by mounting the specimens directly in Canada balsam on glass slides, allowed to dry at room temperature for several days, and identified with the appropriate taxonomic keys [28–30] and with access to the reference collection of Neotropical *Culicoides* housed at the Museo de La Plata, Buenos Aires, Argentina. Voucher specimens of both dipteran groups are available upon request.

### Statistical analyses

Data were statistically analysed for impact of insecticide intervention (abundance and distribution) and climatic variables (temperature and rainfall). Household covariate data, the abundance of people, dogs, and chickens were collected separately from dipteran abundance, being recorded once per round as part of routine trial activities [21]. The per household covariate data recorded on the date closest to that of Diptera capture was assumed to be representative for each household. Data were matched to dipteran counts by household ID and date. To assess the impact of the insecticide interventions, we compared changes in the total numbers (as well as numbers of males + females separately) of mosquitoes and biting midges captured per household, and at each of the described house, dog, and chicken capture sites. The abundance and distribution of both dipteran groups inside houses, dog, and chicken sites was based on the C-arm as it is a better representative of the natural dispersion compared to the treatment arms.

Daily Diptera trapping records per household were excluded from analysis where any dipteran group (mosquitoes or *Culicoides*) or trap location within households were missing. Similarly, data were also excluded if household covariate data were missing. Outliers, such as households associated with unusually high host abundance (> 1000 chickens such as chicken farms) were also excluded from analyses.

Being highly over-dispersed, dipteran counts were analysed by negative binomial regression. Household host abundances of humans, dogs, and chickens plus seasonal variation between rounds were expected to confound capture rates of biting Diptera, thus, we adjusted for these by inclusion trapping round and host abundance as fixed *a priori* predictors in all Diptera count analyses. Finally, repeated sampling across municipalities and within some households led to important structuring in the data. This was accounted for in all multivariate models by clustering on the highest level of structuring, municipality [31].

Raw monthly data of the control arm were used to plot monthly pattern of capture rate over climatic variables as without the intervention effects it was considered to be the most indicative of seasonal trends. The 3-day average temperature/rainfall associated with each Diptera trapping day were used for the statistical analysis, confirming any association between Diptera count on a given day and local climate variables. Climatic plots were constructed using Geometric-Williams (GW) means plus 95% CI to make a fairer comparison due to overdispersion over daily Diptera capture rates. All data were analysed in STATA v.15 (StataCorp LP, College Station, TX, USA).

## Results

In total, 14,609 blood-feeding dipterans were collected during the sampling period (Table 1), consisting of 3145 mosquitoes (64.2% females and 35.8% males) (Table 2) and 11,464 biting midges (92.8% females and 7.2% males) (Table 3). Mosquitoes were collected in 77% of all households (1–10 specimens = 73.6%; 10–100 specimens = 25.1%; 100–1000 specimens = 1.3%), and biting midges were collected in 79% of all households (1–10 specimens = 49.8%; 10–100 specimens = 39.1%; 100–1000 specimens = 10.4%; ≥ 1000 specimens = 0.7%). In total, 345 observations of mosquitoes and midges from 1035 trapping days, and 280 houses were recorded in all 42 study clusters (Table 1). Apart from sand flies [18, 21], no other haematophagous Diptera were captured in sufficient numbers to be considered in this study.

**Table 1:** Summary of trapping effort, numbers captured and distribution of Culicidae and *Culicoides*

Dipteran group	Trial Arm	Proportion of positive trap days	Proportion of positive households	Total no. of specimens	House site	Dog sleeping site	Chicken roosting site
Culicidae	C	0.81 (100/123)	0.82 (84/103)	1372	517	280	575
	PI	0.72 (81/110)	0.73 (66/88)	658	231	190	237
	DC	0.76 (85/112)	0.78 (69/89)	1115	621	169	325
	Total	0.76 (266/347)	0.77 (219/282)	3145	1369	639	1137
*Culicoides*	C	0.78 (96/123)	0.81 (83/103)	4803	117	620	4066
	PI	0.71 (80/110)	0.77 (69/88)	2986	78	290	2618
	DC	0.77 (86/112)	0.81 (72/89)	3675	162	486	3027
	Total	0.75 (262/347)	0.79 (224/282)	11,464	357	1396	9711

*Abbreviations*: C, control-arm; PI, pheromone + lamda-cyhalothrin insecticide-arm; DC**,** deltamethrin dog-collar-arm

**Table 2:** Culicidae captured between July 2015 and April 2016 with CDC-light traps in households from the municipalities of the Araçatuba (São Paulo, Brazil) study area. Numbers summed across all arms

Taxon	♂	♀	♂ + ♀	%
***Culex***	1044	1710	2754	87.6
*Aedeomyia*	11	94	105	3.3
*Anopheles*	24	67	91	2.9
*Mansonia*	28	52	80	2.5
***Aedes***	9	28	37	1.2
*Coquillettidia*	4	28	32	1.0
*Uranotaenia*	1	32	33	1.0
* Psorophora*	1	2	3	< 0.1
*Sabethes*	1	1	2	< 0.1
Damaged or unidentified	2	6	8	0.2
Total	1125	2020	3145

**Table 3:** *Culicoides* species captured between July 2015 and April 2016 with CDC-light traps in households from the municipalities of the Araçatuba (São Paulo, Brazil) study area summed across all arms

Species	♂	♀	♂ + ♀	%
*C. leopoldoi*	311	6740	7057	61.5
*C. limai*	314	1563	1877	16.4
*C. insignis*	72	1391	1463	12.8
*C. venezuelensis*	31	294	325	2.8
*C. pusillus*	29	220	249	2.2
*C.* cf*. filarifer*^a^	29	176	205	1.8
*C. lutzi*	10	132	142	1.3
*C. foxi*	16	90	106	0.9
*C. debilipalpis*	1	12	13	0.1
*C. paraensis*	1	7	8	< 0.1
*C. fernandoi*	1	4	5	< 0.1
*C. gavaldoni*	0	2	2	< 0.1
*C. peruvianus*	0	1	1	< 0.1
*Culicoides* sp. 1^b^	0	1	1	< 0.1
*Culicoides* sp. 2 ^b^	0	1	1	< 0.1
Damaged	1	8	9	< 0.1
Total	816	10,648	11,464	

^a^*Culicoides* cf. *filarifer* includes a group of nearly indistinguishable species with consistent morphological features of *Cu. filarifer* and/or *Cu. ocumarensis*

^b^Insufficient numbers of *Culicoides* spp. 1 and 2 impeded species determination

### Mosquitoes (Culicidae)

#### Species richness

Nine genera of the Culicidae were trapped during this study (Table 2). *Culex* was the most abundant genus and comprised 2754 specimens (87.6% of the catches), followed by *Aedeomyia* (105, 3.3%), while *Anopheles*, *Aedes* and *Mansonia* contributed < 7% (Table 2). According to the 30% of subsampled males, *Cx. quinquefasciatus* was the most frequently occurring species (*n* = 219, 69.7%) followed by *Cx. coronator* (*n* = 34, 11.3%), *Cx. bidens* (*n* = 12, 4.0%), *Cx. nigripalpus* (*n* = 8, 2.6%), *Cx. chidesteri* (*n* = 7, 2.3%), and at least three other *Culex* unidentified species (*n* = 10, 3.3%). *Culex quinquefasciatus* specimens were present in similar proportions at the three trap locations (house, dog sleeping site and chicken site). The remaining specimens belonged to the group of *Culex* subgenus *Melanoconion* (*n* = 24; 7.6%). *Aedeomyia squamipennis* was the second most abundant species (*n* = 105, 3.3%). Within the genus *Anopheles*, *An. triannulatus* accounted for most catches (*n* = 14, 58.3%) and the remainder (*n* = 10, 42.7%) belonged to other species within the subgenus *Nyssorhynchus*. At least four species of *Mansonia* were found: *Ma. humeralis*, *Ma. titillans*, *Ma. fonsecai* and one unidentified species (Table 2). The genus *Aedes* was represented by *Ae. aegypti*, *Ae. albopictus*, *Ae. serratus* and another unidentified species. Three species of the genus *Coquillettidia* were found: *Co. venezuelensis*, *Co. nigripalpus* and another unidentified species. A few specimens of other genera were also occasionally recorded (Table 2).

#### Abundance and distribution in the C-arm

**Fig. 2:**
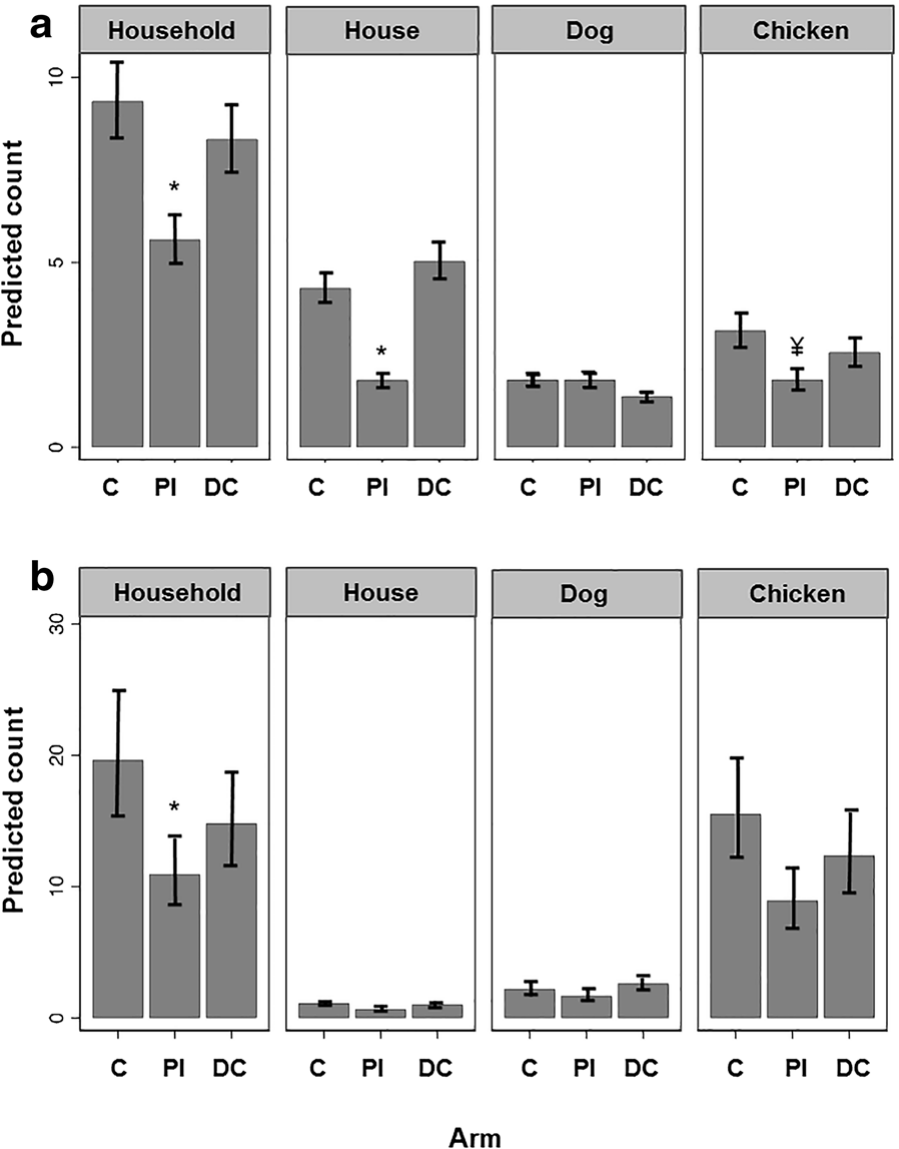
Predicted mean count (95% CI) of both groups of biting Diptera studied. Culicidae (**a**) and *Culicoides* (**b**) at household level and in the three trap locations (house, dog, chicken) in the three intervention arms (C, control; PI, pheromone + lamba-cyhalothrin; and DC, deltamethrin dog-collar). Statistical differences ^¥^*P* < 0.1, *P < 0.05 and are with respect to control trap catches within each trapping location. The analysis takes into account all covariate data and modelled effect

Mosquitoes (1372) were mostly collected at chicken roosting sites (*n* = 575, 41.9%), closely followed by traps located inside houses (*n* = 517, 37.7%) and in smaller numbers in traps near dog sleeping sites (*n* = 280, 20.4%) (Table 1, Fig 2a). Nine genera were recorded in chicken roosting sites, 8 in dog sleeping sites, and 7 in houses.

#### Annual dynamics and climatic variables

**Fig. 3:**
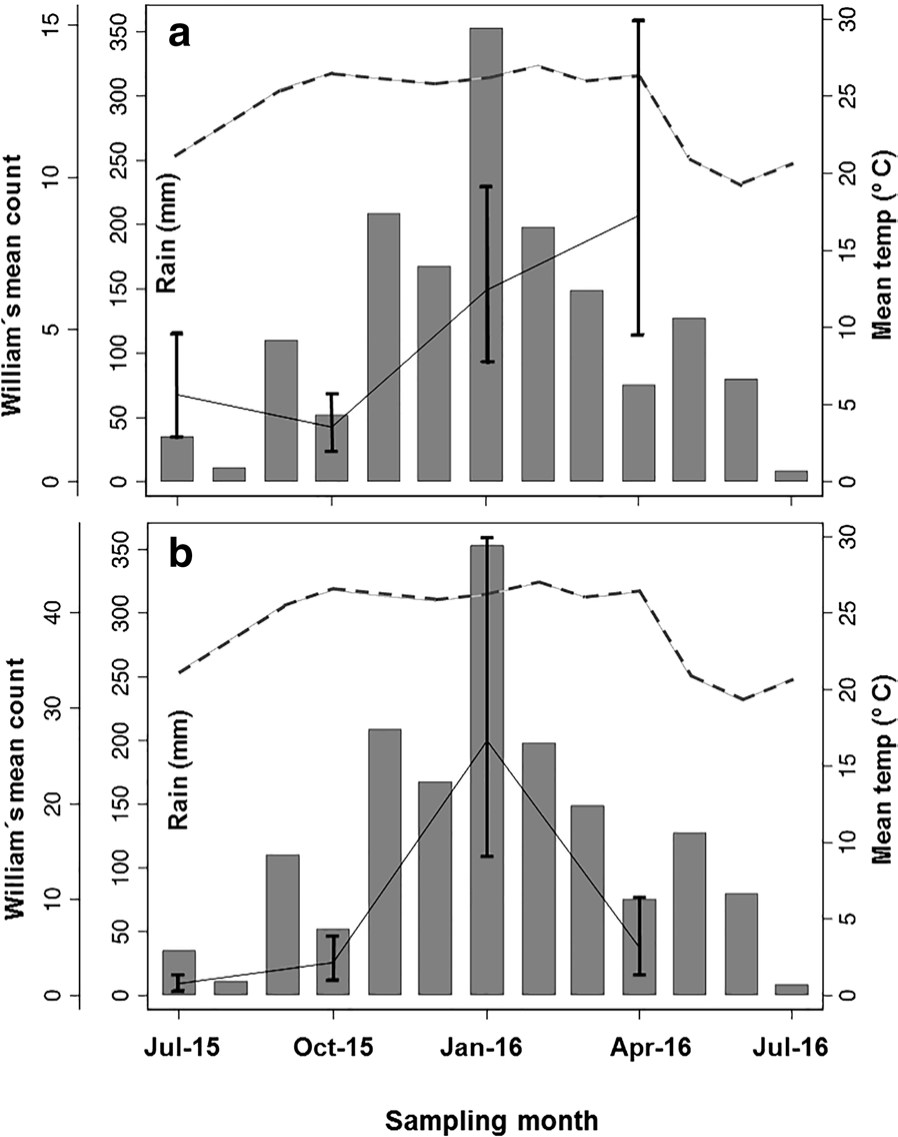
Monthly seasonal occurrence of both biting Diptera groups studied. Culicidae (**a**) and *Culicoides* (**b**) recorded with CDC-light traps in four sampling periods from July 2015 to July 2016 (showed as median trap date per round) in the control arm of the mesoregion of Araçatuba (São Paulo, Brazil). Rainfall (mm) is represented by vertical dark bars, mean temperature (°C) by upper discontinuous grey line and catches (Geometric-William means ± CI) are represented by a continuous black line

Mosquitoes were predominantly captured during the summer and early autumn (January and April 2016, rounds 16 and 17, respectively) and to a lesser extent in the early winter and spring (July and October 2015, rounds 14 and 15, respectively). The average daily temperature had a significant positive effect on the average number of mosquitoes (*Z* = 1.97, *P* = 0.049) with a 0.10 factor change per degree increase in temperature. Rainfall average did not significantly affect mosquito abundance (*Z* = 0.78, *P* = 0.437) (Fig. 3a). Up to 4× times more specimens were captured in April (the most abundant, geometric mean (GM) = 7.9; 15.0–4.8) compared to October (the poorest, GM = 2.3; 3.1–1.8). Similar annual variation was seen in all the captured genera, peaking in summer-autumn (Additional file 2: Figure S1).

#### Impact of insecticide interventions on mosquito abundance and distribution

Analysis of mosquito abundances (females + males) revealed a significant reduction (56%) in the PI-arm in the household traps (chicken roosting sites + dog sleeping sites + houses compared to the controls) (IRR = 0.54, 95% CI: 0.30–0.97, *P* = 0.04). There were significant reductions in those sites where they were most commonly caught, i.e. in houses (IRR = 0.39, 95% CI: 0.20–0.74, *P* ≤ 0.01) and at the chicken roosting sites, although the latter only reached borderline significance (IRR = 0.52, 95% CI: 0.25–1.07, *P* = 0.08) (Table 4, Fig. 2a). Analysis of female numbers alone showed that they followed a similar pattern with significant reductions overall at the household level (IRR = 0.49, CI: 0.25–0.96, *P* = 0.04) and also at chicken roosting sites (IRR = 0.40, 95% CI: 0.18–0.86, *P* = 0.02) and in houses (IRR = 0.42, 95% CI: 0.21–0.85, *P* = 0.02) (Additional file 3: Table S2).

**Table 4:** Summary of the intervention effects (IRR (95% CIs)) on Culicidae and *Culicoides* at the household level and at the three trap positions (house, dog and chicken) compared to control (placebo)

	Variable			Trap position		
			Household Total	House	Dog	Chicken
Culicidae	Arm	PI	0.54 (0.30–0.97)*	0.39 (0.20–0.74)*	0.88 (0.42–1.85)	0.52 (0.25–1.07)^¥^
		DC	0.94 (0.55–1.59)	1.19 (0.60–2.33)	0.81 (0.43–1.5)	0.86 (0.42–1.79)
	Round	15	0.67 (0.38–1.15)	0.74 (0.37–1.49)	0.39 (0.2–0.78)*	0.74 (0.35–1.56)
		16	2.67 (1.4–5.09)*	2.47 (1.05–5.78)*	1.21 (0.54–2.69)	4.88 (2.2–10.84)***
		17	2.22 (0.91–5.41)^¥^	2.38 (0.85–6.64)¥	1.10 (0.51–2.41)	3.68 (1.24–10.93)*
	Host	H	1.0 (0.90–1.12)	0.96 (0.84–1.11)	1.02 (0.93–1.12)	1.03 (0.92–1.16)
		D	0.98 (0.89–1.08)	1.05 (0.93–1.2)	0.94 (0.85–1.04)	0.98 (0.86–1.11)
		C	1.01 (1–1.02)^¥^	1.01 (0.99–1.02)	1.01 (0.99–1.03)	1.01 (0.99–1.02)
*Culicoides*	Arm	PI	0.47 (0.26–0.85)*	0.54 (0.2–1.47)	0.64 (0.33–1.24)	0.48 (0.27–0.84)*
		DC	0.74 (0.40–1.37)	0.94 (0.27–3.32)	1.29 (0.72–2.3)	0.78 (0.43–1.4)
	Round	15	3.15 (1.8–5.51)***	2.04 (0.49–8.59)	3.40 (1.72–6.75)***	3.48 (1.48–8.19)*
		16	31.6 (19.4–51.6)***	4.16 (1.02–17.02)*	42.37 (21.56–83.23)***	37.8 (20.2–70.4)***
		17	13.32 (6.9–25.3)***	1.59 (0.38–6.64)	20.18 (9.06–44.92)***	15.6 (7.39–32.9)***
	Host	H	0.95 (0.86–1.05)	0.88 (0.78–0.98)*	1.02 (0.94–1.1)	0.94 (0.83–1.06)
		D	1.13 (1.03–1.25)*	0.97 (0.83–1.13)	1.07 (0.97–1.18)	1.16 (1.04–1.29)*
		C	1.01 (1.0–1.02)*	1.01 (1.0–1.02)*	1.01 (1.0–1.03)^¥^	1.01 (1.0–1.02)*

*Abbreviations*: Arm, treatment arm; PI, pheromone + lambda-cyhalothrin insecticide; DC, deltamethrin dog-collar. Hosts; H = human, D = dog, C = chicken. Categorical variables (control arm and round 14) were used as references for the comparisons. ^¥^*P* ≤ 0.1, *P < 0.05, ***P < 0.001. Intervention effects were estimated from negative binomial regression outcome of total capture rates (females + males) for each Dipteran group. This analysis takes into account the effect of *a priori* predictors, factor change in capture rate [IRR (95% CIs)] and clustering on municipality

The insecticidal collars did not have a significant impact on capture rates of mosquitoes at any of the household sites compared to untreated collars in the C-arm (Table 4, Fig. 2a).

Rounds 16 and 17 showed significant peaks of mosquito abundance compared to round 14 (Table 4). The number of chickens per roost was significantly associated with household level mosquito capture in all treatment arms, however, the effect was small with only a 0–2% increase in mosquito capture rate per additional chicken (IRR = 1.0, CI: 1.00–1.02, *P* = 0.08) (Table 4).

### Biting midges (*Culicoides*)

#### Species richness

A total of 15 *Culicoides* species were captured. *Culicoides leopoldoi* was the most abundant species (*n* = 7057 specimens, 61.5%), followed by *C. limai* (*n* = 1877; 16.4%), and *C. insignis* (*n* = 1463; 12.8%). Small numbers of 12 other species accounted for less than 10% of the total captured (Table 3).

#### Abundance and distribution in the C-arm

*Culicoides* (*n* = 4803 specimens) were trapped most frequently at chicken roosting sites (*n* = 4066, 84.7%), followed by dog sleeping sites (*n* = 620, 12.9%), and to a minor extent in houses (*n* = 117, 2.4%) (Table 1). Thirteen species were recorded in chicken roosting sites and 11 in both dog sleeping sites and in houses.

#### Annual dynamics and climatic variables

Adult *Culicoides* were very abundant during the warmest and wettest summer sampling months (January 2016; round 16). By comparison numbers collected in autumn (April 2016; round 17), winter (July 2015; round 14) and spring (October 2016; round 15) were much lower. The average daily temperature had a positive marginal effect on the average numbers of mosquitoes (*Z* = 1.71, *P* = 0.087) with a 0.17 factor increase per degree increase in temperature. There was no significant relationship between rainfall average and *Culicoides* abundance (*Z* = 0.25, *P* = 0.802). The number of *Culicoides* trapped was much greater (14×) in January (GM = 25.2, 48.4–12.2) compared to July, which had the lowest catch (GM = 1.7, 1.9–1.3) (Fig. 3b).

Differences in abundance of the three dominant species were observed throughout the year. *Culicoides leopoldoi* was present in substantial numbers throughout all four sampling periods with a peak of abundance in January 2016, whereas *C. limai* was absent in July 2015 but present since October 2015. *Culicoides insignis* was particularly abundant during the rainy season (January-April 2016) but almost absent over the remaining sampling periods. The other 13 less abundant species followed a similar pattern to *C. leopoldoi* (Additional file 2: Figure S1).

#### Impact of the insecticide interventions on Culicoides abundance and distribution

Analysis of *Culicoides* abundance indicated that the use of lambda-cyhalothrin in the PI-arm significantly reduced (53%) the number of *Culicoides* (females + males) across the total of all household captures compared to the control arm (IRR = 0.47, 95% CI: 0.26–0.85, *P* = 0.01) (Table 4, Fig. 2b). However, when the household trap sites were examined individually, only the reduction of *Culicoides* in chicken roosting sites was significant (IRR = 0.48, 95% CI: 0.27–0.84, *P* = 0.01) (Table 4, Fig. 2b). Numbers of females alone followed a similar pattern with a significant reduction at the household level (IRR = 0.45, 95% CI: 0.25–0.81, *P* = 0.01) and at chicken roosting sites (IRR = 0.47, 95% CI: 0.26–0.84, *P* = 0.01) but not in houses or at dog sleeping sites (Additional file 3: Table S2).

The use of deltamethrin-impregnated dog collars in the DC-arm did not significantly alter *Culicoides* capture rates compared to untreated collars at any of the peridomestic sites (Table 4, Fig. 2b).

Rounds 15, 16 and 17 were all associated with a significant increase in *Culicoides* abundance compared to round 14 (Table 4). The abundance of animal hosts was a significant predictor of *Culicoides* capture rates, and greater numbers of both dogs and chickens were associated with larger numbers of *Culicoides* midges (Table 4).

## Discussion

Overall, the pheromone + insecticide intervention applied to control *Lu. longipalpis* in chicken roosting sites resulted in a reduction in the numbers of Culicidae (mosquitoes) (56%) and *Culicoides* (biting midges) (53%) in the peridomestic environment (chicken roosting sites + dog sleeping sites + in houses). By contrast, deltamethrin-impregnated dog collars had no impact on the numbers of either mosquitoes or biting midges. It is likely that the reduction in numbers in the PI-arm was caused by increased mosquito and midge mortality near to chicken roosting sites where insecticide was applied to surfaces that can serve as resting places for blood-seeking/blood-fed dipterans. In addition, the mortality effect of the insecticide around chicken roosting sites led to a reduction of mosquitoes (but not biting midges) in houses. A reduction in *Lu. longipalpis* sand fly abundance attributed to the insecticide + pheromone was also observed in the PI-arm (66% in females and 69% in males) [21]. This was slightly higher than the observed percent reductions in mosquitoes and biting midges.

The insecticide deployment had no significant effect on species richness. A few more species (all minor species < 0.1%) of both dipteran groups were found in traps located near chicken roosting sites than in the other locations, which is perhaps not surprising considering that wild environments are prone to have higher diversity than other sites because they have high host availability, variable vegetation, resting places, and potential breeding sites [32, 33].

Overall, culicids were common and present in most of the sampled households. In particular *Culex* spp. were abundant and represented nearly 90% of the total catches. *Culex quinquefasciatus*, the most commonly collected species, is widely distributed in the equatorial, tropical and subtropical regions of Brazil [3, 34]. This species is highly endophilic and opportunistic and the females might feed on humans, chickens or many other available hosts, i.e. dogs, horses, cattle, rodents, rabbits and pigs [1, 35–37]. The second most abundant genus was *Aedeomyia*, represented by the sole tropical species *Ad. squamipennis*. This species is found throughout most of the Neotropics and is considered to be an important vector of various bird viruses, including Gamboa virus [38]. It is reported as an ornithophilic species, commonly found in association with chickens. Important dengue vectors (*Ae. aegypti* and *Ae. albopictus*) were uncommon because light traps were relatively ineffective to collect these daytime biters, but they were found mostly in traps in houses confirming their preference for feeding primarily on humans and resting indoors [39].

The study also revealed a rich and abundant midge fauna in peridomestic environments and most of the *Culicoides* species that have been reported widely in the Neotropics were recorded here. The most predominant species trapped near chickens was *C. leopoldoi*, a widely-distributed species that is associated with poultry and a wide range of mammals in Brazil [40–42]. *Culicoides limai* is a forest species with a broad host range [40, 43]. Other common species collected such as *C. insignis* and *C. pusillus*, are known to be major and potential vectors of BTV, respectively [9, 44]. *Culicoides insignis* is a widespread species often associated with animals and commonly found in pasture environments with cattle and pigs [42, 45–48] and to a lesser extent attracted towards poultry [41]. In spite of the low numbers collected, the roles of *C. paraensis* involved in OROV and *C. debilipalpis*, another competent BTV vector [49], should be considered in future health surveillance programmes both for their vectorial capacity and annoyance of humans [50].

Most *Culicoides* specimens were captured in outdoor traps; the small proportion trapped indoors, predominantly males, suggested an exophilic behaviour and reluctance to enter buildings to feed on humans. Although studies on the degree of exo/endophagy behaviour of *Culicoides* has not been reported previously in Brazil, it is assumed that most *Culicoides* species in farm environments are exophilic in tropical areas. Consequently, *Culicoides* outdoor activity is presumably associated with the presence of host availability (cattle and poultry) [51]. The high proportion of specimens collected in chicken shelters contrasts to the numbers collected inside human dwellings, supporting the hypothesis that outdoor animals (e.g. chickens) are the primary host preference for bird-associated *Culicoides* species.

Our study found that mosquitoes (mostly *Culex*) were present throughout the year although there was an increase in abundance from summer to early autumn. The relationship between mosquito abundance and meteorological conditions has been extensively reported on by different authors but the seasonality of peak mosquito numbers varies geographically [52]. These variations may be related to the interaction of availability of breeding sites and other unidentified ecological factors [53]. Other studies have reported high densities of *Cx. quinquefasciatus* in areas in which preferential breeding sites are scarce, suggesting the existence other elements related to intrinsic residential characteristics as important factors for maintaining the infestation of this mosquito species.

Although substantially higher numbers of *Culicoides* were present in the rainy season, their abundance was not linked directly to rainfall in contrast to other studies [43, 48, 54, 55]. Our catches also indicated different patterns of seasonal occurrence possibly related to different potential ecological requirements, i.e. water availability or land use. *Culicoides leopoldoi* was captured throughout the entire study period, although it was much more abundant in the rainy season [43]. By contrast, *C. insignis* was restricted to the wet season. This species has previously been captured during autumn and winter in Argentina [56] and during the rainy season in Brazil [48].

Methods to control adult mosquitoes over small areas most commonly include application of insecticide “barrier sprays” on vegetation and other structures where mosquitoes rest during the day [57]. However, mosquito control efficacy with insecticides is highly controversial and success depends on multiple elements [58]. Residual spraying of lambda-cyhalothrin against *Cx. pipiens*, *Ae. albopictus* [57], and *Anopheles* spp. [59] has been carried out in many regions of the world with variable degrees of entomological efficacy. Ground-applied space spray applications to control *Culex* and *Aedes* mosquitoes tend not be effective, partially because they tend to rest indoors on objects and other structures that are inaccessible or should not be sprayed (e.g. personal items) rather than on walls and ceilings [59, 60]. Interestingly, our study showed that long-term insecticide spraying of poultry shelters targeted adult mosquito (*Culex*) resting sites and reduced the numbers found in human dwellings as a collateral effect.

There are few published evaluations of the impact of insecticide spraying in houses, animal shelters or poultry on *Culicoides* abundance*.* Most studies have focused on topical insecticide applications to livestock or physical barriers to improve animal welfare by population suppression of biting midges [61, 62]. The impact of environmental spraying in and around sheep pens against *Culicoides* in Europe was also assessed against BTV transmission although the results were not conclusive [63, 64]. The insecticide lambda-cyhalothrin has both repellent and adulticide action against *Culicoides* spp. [62, 65]; other organophosphates and pyrethroids have historically been evaluated against *Culicoides* with overall unsuccessful results in field trials [8, 63]. Thus, the results presented in the present study are promising. The impact of insecticide could be further enhanced if used against adult resting sites and larval feeding sites [66]; in one recent study, a combination of adult insecticides applied outdoors on walls and roofs of animal shelters, combined with applying larvicides on *Culicoides* breeding sites, resulted in significant reductions in *Culicoides* abundance [67].

Our study suggested that Scalibor dog collars do not offer any protection against biting Diptera populations. Deltamethrin-impregnated collars have provided anti-feeding protection or insecticidal effects against mosquitoes (e.g. *Culex pipiens pipiens*) for up to six months under laboratory trials [68], making this device potentially an effective solution against common dirofilariasis given the proven feeding behaviour of *Culex* on dogs [69]. However, our results did not demonstrate effectiveness in reducing mosquito numbers. Similar experiments to test insecticide-impregnated collars against bites of *Culicoides* have not been reported perhaps because *Culicoides* do not readily feed on dogs [70, 71] supported also by the overall lack of major pathogen transmission (e.g. African horse sickness virus) [72].

We attribute the reductions in the abundance of biting dipterans in the PI-arm to the residual activity of the insecticide sprayed at the chicken roosting sites, which are a likely blood source for both biting Diptera groups, and not to an additional effect of the synthetic sand fly pheromone because the sex-aggregation pheromone is species specific for *Lu. longipalpis* it would only attract that species [22]. It is unsurprising that the reductions in the biting dipterans was related to the presence of insecticide; the current analyses demonstrate the potential additional benefit of such insecticidal interventions against sand flies [21] and other vectors (i.e. *Culicoides* and mosquitoes) of other important diseases. Such benefits will depend on the behaviour of the given Dipteran species, which may vary in their degree of zoophily and thus their likelihood of coming into contact with the insecticide.

## Conclusions

To the best of our knowledge, our study represents the first promising large-scale attempt to control poultry biters in peridomestic environments of Latin America. This study demonstrates that spraying lambda-cyhalothrin has a beneficial effect against medically important adult dipteran populations in and around poultry roosts. From a vector control perspective, this intervention seems likely to be an effective control measure to reduce blood-feeding dipterans and thus, the feeding pressure and capacity to spread pathogens (other than *Le. infantum*), which present a substantial impact on poultry. The effect of any sustained insecticide spraying campaigns in triggering insecticide resistance and the environmental consequences on beneficial non-target insects, such as dung beetles and pollinators, warrants further investigation.

## Supplementary information

**Additional file 1: Table S1. **Summary of raw data counts by municipality of Culicidae and *Culicoides* in the three intervention arms. *Abbreviations*: C, control; PI, pheromone + lambda-cyhalothrin insecticide spraying; DC, deltamethrin dog-collar in the mesoregion of Araçatuba (São Paulo State, Brazil).

**Additional file 2: Figure S1. **Monthly seasonal occurrence of the most frequently trapped mosquito and midge genera. Culicidae (**a**) and *Culicoides* (**b**) during the four sampling periods (July 2015, October 2015, January 2016 and April 2016) in the mesoregion of Araçatuba (São Paulo State, Brazil).

**Additional file 3: Table S2.** Summary of the intervention effects on female Culicidae and *Culicoides* at the household level and at the three trap positions (house, dog sleeping site and chicken roosting site compared to control (placebo). *Abbreviations*: Arm, treatment arm; PI, pheromone + lambda-cyhalothrin insecticide spraying; DC, deltamethrin impregnated dog-collar. Categorical variables (arm = control and round = 14) were used as reference for comparison. ^¥^*P* ≤ 0.1, * *P *≤ 0.05, *** *P *≤ 0.001, Intervention effects were estimated from negative binomial regression outcome of total capture rates (females) for each dipteran group. This analysis takes into account the effect of *a priori* predictors, factor change in capture rate [IRR (95% CIs)] and clustering on municipality.

## Data Availability

The essential data are contained in the manuscript and its additional files. Raw data that support the findings of this study are available upon request.

## Abbreviations

RCT: Randomised control trial
OROV: Oropouche virus
BTV: Bluetongue virus
VL: Visceral leishmaniasis
C: Control-arm
PI: Pheromone + lambda-cyhalothrin insecticide-arm
DC: Deltamethrin dog-collar-arm
