# Supplementary material for: Significant reduction in abundance of peridomestic mosquitoes (Culicidae) and Culicoides midges (Ceratopogonidae) after chemical intervention in western São Paulo, Brazil
Source: Parasit Vectors. 2020 Nov 7;13:549. doi: 10.1186/s13071-020-04427-1 (PMC7648319; doi:10.1186/s13071-020-04427-1)
Supplement: Supplementary file 1 — Additional file 1: Table S1. Summary of raw data counts by municipality of Culicidae and Culicoides in the three intervention arms. Abbreviations: C, control; PI, pheromone + lambda-cyhalothrin insecticide spraying; DC, deltamethrin dog-collar in the mesoregion of Araçatuba (São Paulo State, Brazil). [file 13071_2020_4427_MOESM1_ESM.docx]

**Additional file 1: Table S1.** Summary of raw data counts by municipality of Culicidae and *Culicoides* in the three intervention arms. *Abbreviations*: C, control; PI, pheromone + lambda-cyhalothrin insecticide spraying; DC, deltamethrin dog-collar in the mesoregion of Araçatuba (São Paulo State, Brazil).

| **Arm** | **Municipality** | **Culicidae** | ***Culicoides*** |
| --- | --- | --- | --- |
| C | Andradina | 58 | 595 |
| C | Araçatuba (3 districts) | 336 | 166 |
| C | Braúna | 78 | 27 |
| C | Clementina | 4 | 37 |
| C | Nova Luzitânia/Lourdes | 707 | 53 |
| C | Penápolis | 101 | 49 |
| C | Pereira de Barreto | 55 | 55 |
| C | Rinópolis | 1792 | 166 |
| C | Santo Antônio de Aracanguá | 53 | 16 |
| C | Santópolis do Aguapeí | 402 | 17 |
| C | Sud Mennucci | 1201 | 148 |
| C | Valparaíso | 16 | 43 |
| PI | Alto Alegre | 63 | 10 |
| PI | Araçatuba (2 districts) | 34 | 104 |
| PI | Auriflama | 345 | 98 |
| PI | Bilac | 1441 | 104 |
| PI | Castilho | 281 | 81 |
| PI | Coroados | 99 | 27 |
| PI | Guararapes | 3 | 56 |
| PI | Mirandopolis | 71 | 59 |
| PI | Murutinga do Sul | 80 | 27 |
| PI | Piacatu | 555 | 85 |
| PI | Sao Jose | 14 | 7 |
| DC | Araçatuba (5 districts) | 172 | 235 |
| DC | Avanhandava | 677 | 136 |
| DC | Barbosa | 764 | 370 |
| DC | Bento de Abreu | 334 | 49 |
| DC | Birigui | 30 | 30 |
| DC | Glicério | 49 | 18 |
| DC | Guaiçara | 63 | 14 |
| DC | Guaraçaí | 561 | 48 |
| DC | Lavinia | 738 | 85 |
| DC | Rubiácea | 61 | 7 |
| DC | Salmourão | 226 | 123 |
