# Supplementary figures and images for: Significant reduction in abundance of peridomestic mosquitoes (Culicidae) and Culicoides midges (Ceratopogonidae) after chemical intervention in western São Paulo, Brazil
Source: Parasit Vectors. 2020 Nov 7;13:549. doi: 10.1186/s13071-020-04427-1 (PMC7648319; doi:10.1186/s13071-020-04427-1)

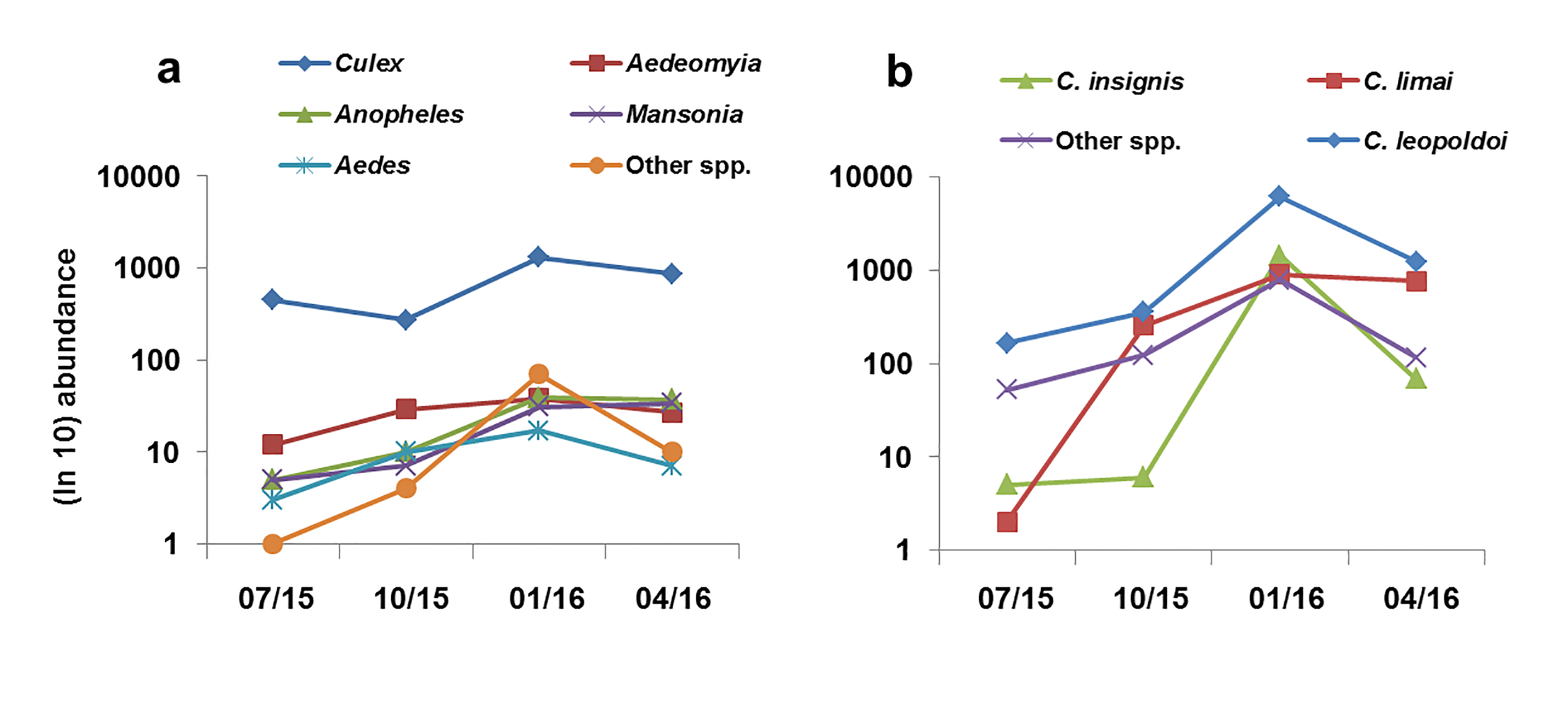

Supplement: Supplementary file 2 — Additional file 2: Figure S1. Monthly seasonal occurrence of the most frequently trapped mosquito and midge genera. Culicidae (a) and Culicoides (b) during the four sampling periods (July 2015, October 2015, January 2016 and April 2016) in the mesoregion of Araçatuba (São Paulo State, Brazil). [file 13071_2020_4427_MOESM2_ESM.tif]
