# Supplementary material for: Significant reduction in abundance of peridomestic mosquitoes (Culicidae) and Culicoides midges (Ceratopogonidae) after chemical intervention in western São Paulo, Brazil
Source: Parasit Vectors. 2020 Nov 7;13:549. doi: 10.1186/s13071-020-04427-1 (PMC7648319; doi:10.1186/s13071-020-04427-1)
Supplement: Supplementary file 3 — Additional file 3: Table S2. Summary of the intervention effects on female Culicidae and Culicoides at the household level and at the three trap positions (house, dog sleeping site and chicken roosting site compared to control (placebo). Abbreviations: Arm, treatment arm; PI, pheromone + lambda-cyhalothrin insecticide spraying; DC, deltamethrin impregnated dog-collar. Categorical variables (arm = control and round = 14) were used as reference for comparison. ¥P ≤ 0.1, * P ≤ 0.05, *** P ≤ 0.001, Intervention effects were estimated from negative binomial regression outcome of total capture rates (females) for each dipteran group. This analysis takes into account the effect of a priori predictors, factor change in capture rate [IRR (95% CIs)] and clustering on municipality. [file 13071_2020_4427_MOESM3_ESM.docx]

**Additional file 3: Table S2.** Summary of the intervention effects (IRR (95% C.I.s.))_on female Culicidae and *Culicoides* at the household level and at the three trap positions (house, dog sleeping site and chicken roosting site compared to control).

|  | **Variable** | | **Total** | **Trap position** | | |
| --- | --- | --- | --- | --- | --- | --- |
|  |  | | **Household** | **House** | **Dog** | **Chicken** |
| **Culicidae** | **Arm** | **PI** | 0.49 (0.25-0.96)* | 0.42 (0.21-0.8)* | 0.72 (0.33-1.6) | 0.4 (0.18-0.86)* |
|  |  | **DC** | 0.76 (0.43-1.37) | 1.02 (0.47-2.22) | 0.69 (0.36-1.33) | 0.7 (0.35-1.41) |
|  | **Round** | **15** | 0.57 (0.33-0.99)* | 0.56 (0.26-1.22) | 0.49 (0.23-1.05)^¥^ | 0.52 (0.26-1.04)^¥^ |
|  |  | **16** | 2.23 (1.16-4.29)* | 1.97 (0.85-4.57) | 1.03 (0.45-2.36) | 3.59 (1.64-7.86)* |
|  |  | **17** | 2.12 (0.84-5.33) | 2.79 (1.01-7.6)* | 1.01 (0.43-2.38) | 2.69 (0.92-7.87)^¥^ |
|  | **Host** | **H** | 0.99 (0.88-1.1) | 0.95 (0.81-1.12) | 1.02 (0.93-1.12) | 1.03 (0.91-1.16) |
|  |  | **D** | 0.97 (0.89-1.06) | 1.03 (0.91-1.17) | 0.94 (0.84-1.04) | 0.96 (0.85-1.08) |
|  |  | **C** | 1.01 (1.0-1.03)* | 1.01 (1.0-1.02)¥ | 1.01 (0.99-1.04) | 1.01 (1-1.03)^¥^ |
| ***Culicoides*** | **Arm** | **PI** | 0.45 (0.25-0.81)* | 0.43 (0.15-1.21) | 0.61 (0.29-1.27) | 0.47 (0.26-0.84)* |
|  |  | **DC** | 0.69 (0.37-1.31) | 0.83 (0.23-3.04) | 1.1 (0.58-2.07) | 0.75 (0.41-1.37) |
|  | **Round** | **15** | 2.82 (1.61-4.94)*** | 1.41 (0.37-5.42) | 4.47 (1.79-11.13)* | 3.12 (1.31-7.44)* |
|  |  | **16** | 30.75 (18.23-51.86)*** | 4.08 (0.95-17.4)¥ | 46.35 (17.97-119.5)*** | 36.21 (19.16-68.4)*** |
|  |  | **17** | 12.34 (6.22-24.48)*** | 1.47 (0.35-6.22) | 21.61 (7.3-64)*** | 14.22 (6.62-30.55)*** |
|  | **Host** | **H** | 0.95 (0.85-1.05) | 0.9 (0.81-0.99)* | 1 (0.93-1.07) | 0.94 (0.83-1.06) |
|  |  | **D** | 1.14 (1.03-1.26)* | 0.95 (0.81-1.12) | 1.08 (0.97-1.21) | 1.16 (1.03-1.3)* |
|  |  | **C** | 1.01 (1-1.02)* | 1.01 (1-1.02)^¥^ | 1.01 (1-1.03)* | 1.01 (1-1.02)* |

*Abbreviations*: Arm, treatment arm; PI, pheromone + lambda-cyhalothrin insecticide spraying; DC, deltamethrin impregnated dog-collar. Categorical variables (arm = control and round = 14) were used as reference for comparison. H = human, D = dog, C = control. ^¥^*P* ≤ 0.1, **P* ≤ 0.05, ****P* ≤ 0.001. Intervention effects were estimated from negative binomial regression outcome of total capture rates (females) for each dipteran group. This analysis takes into account the effect of *a priori* predictors, factor change in capture rate [IRR (95% CIs)] and clustering on municipality.
